# Development of the TabacoQuest app for computerization of data collection on smoking in psychiatric nursing

**DOI:** 10.1590/1518-8345.0661.2726

**Published:** 2016-08-29

**Authors:** Renata Marques de Oliveira, Alexandre Freitas Duarte, Domingos Alves, Antonia Regina Ferreira Furegato

**Affiliations:** 1 Doctoral Student, Escola de Enfermagem de Ribeirão Preto, Universidade de São Paulo, PAHO/WHO Collaborating Centre for Nursing Research Development, Ribeirão Preto, SP, Brazil.; 2 BSc in Biomedical Informatics, Faculdade de Filosofia, Ciências e Letras de Ribeirão Preto, Faculdade de Medicina de Ribeirão Preto, Universidade de São Paulo, Ribeirão Preto, SP, Brazil.; 3 PhD, Assistant Professor, Faculdade de Medicina de Ribeirão Preto, Universidade de São Paulo, Ribeirão Preto, SP. Brazil.; 4 PhD, Retired Full Professor, Escola de Enfermagem de Ribeirão Preto, Universidade de São Paulo, PAHO/WHO Collaborating Centre for Nursing Research Development, Ribeirão Preto, SP, Brazil.

**Keywords:** Software, Nursing Informatics, Mental Health, Psychiatric Nursing

## Abstract

**Objective:**

to develop a mobile app for research on the use of tobacco among psychiatric patients and the general population.

**Method:**

applied research with the technological development of an app for data collection on an Android tablet. For its development, we considered three criteria: data security, benefits for participants and optimization of the time of researchers. We performed tests with twenty fictitious participants and a final test with six pilots.

**Results:**

the app collects data, stores them in the database of the tablet and export then to an Excel spreadsheet. Resources: calculator, stopwatch, offline operation, branching logic, field validation and automatic tabulation.

**Conclusion:**

the app prevents human error, increases the quality of the data by validating them during the interview, allows the performing of automatic tabulation and makes the interviews less tiring. Its success may encourage the use of this and other computational resources by nurses as a research tool.

## Introduction

Research studies in mental health are essential for the planning of the care, the organization of health services, the development of public health policies and, consequently, the improvement of the quality of life of patients^1^.

Studies suggest that persons with mental disorders feel pleased to participate as volunteers in research studies, since they believe that they can help individuals who go through experiences similar to them, and also bring benefits to themselves (therapeutic effect of disclosing their experiences)^1^ ^-^ ^2^.

On the other hand, psychiatric patients may present difficulties because of changes in mental, status such as anhedonia, inattention, difficulty with memory, lack of concentration, anxiety, among others. After an interview on suicide, seventy-nine schizophrenic individuals reported their experiences as participants of the research. They complained they had experienced anxiety, fatigue, difficulty with memory, stress, restlessness and nervousness^1^.

To minimize the discomfort of the respondent, computerized data collection has been used (Mobile Computer Assisted Personal Interviewing - MCAPI), using apps and software products, developed for smartphones and tablets, with digital, attractive, dynamic and interactive questionnaires that motivate participation in the study^3^ ^-^ ^5^. It is common in human beings to feel anxious during an interview. By transferring the attention to the digital questionnaire, the respondent is no longer the main focus, what helps him or her cope with the situation. This highlights the usefulness of MCAPI for all groups of the population and its importance in the different areas of research. With psychiatric patients, however, the MCAPI has a particular importance because the anxiety of the respondent is added to the particular difficulties of other changes in mental state, specific to mental disorders. It is believed that digital questionnaires, by allowing more interaction during the interview, can stimulate psychiatric patients who feel unmotivated to participate in research and promote the quality of the answers by increasing the attention/concentration and decreasing anxiety and weariness. 

The rapid spread of mobile devices in the market has favored the integration of this technology in the area of health, both in research studies and in care assistance and management. In a Swedish study, with 398 nurses and nursing students, it was found that most believed that mobile devices can benefit nursing efforts, without prejudice to the quality of the care and increasing the confidence of patients in the professionals^6^. It is believed that this increased confidence can be extended to researchers. 

In addition to the benefits to the respondent, the computerized data collection decreases the possibility of human error during registration of the answers, contributing to a better quality and security of the data and increasing the scientific credibility of the research results^3^ ^,^ ^7^ ^-^ ^9^.

The MCAPI has been introduced in epidemiological research studies. The Brazilian Institute of Geography and Statistics (IBGE) was awarded by the United Nations Educational, Scientific and Cultural Organization (UNESCO) for the first computerized national census which ensured quality of data, in addition to economic and environmental advantages.

The use of tobacco by psychiatric patients is a subject that has been highlighted in scientific research by portraying a serious public health problem. While the prevalence of smokers in the world population is approximately 20%, in some groups of psychiatric patients, particularly among schizophrenic ones, it can reach approximately 85%^10^. It encourages researchers to investigate the subject, with the possibility of surveying a variety of variables.

Faced with the need to interview psychiatric patients and the general population for a cross-sectional epidemiological study on smoking, with a large number of variables, we considered the possibility of computerizing the questionnaires, in view of its benefits for research participants and for the quality of the data. 

Although we found no studies on the development of this type of app for data collection, on the use of tobacco among psychiatric patients, we believe that it is relevant because it is more dynamic, attractive and interactive than paper-based research studies. 

This study aimed to develop a mobile app for research on the use of tobacco among psychiatric patients and the general population.

## Methods

This is an applied research with the technological development of a app for computerized data collection on an mobile device. The applied research is characterized by its practical utility, as it is intended for the creation of resources that can help solve identified problems. The app was developed for the doctoral dissertation "Prevalence and profile of smoking among persons with mental disorders and the general population", of the Graduate Program in Psychiatric Nursing of the Nursing School of Ribeirão Preto, University of São Paulo, Brazil.

The proposal in this study on smoking, approved by the Ethics Committee (Nursing School of Ribeirão Preto - EERP - 603/873-0, CAAE 21101113.3.3001.5413, was to conduct interviews with 378 participants, treated in three health services of a city in the State of São Paulo: mental health clinic (n=126), psychiatric hospital (n=126) and basic health unit (n=126). We provided for the implementation of nine questionnaires related to the topic of the study: 1) Questionnaire for the identification of persons who attend mental health services and primary health care (prepared specially for the study), 2) Brazilian Economic Classification Criterion - CCEB-2014, 3) Suicide risk monitoring scale, 4) Brief Psychiatric Rating Scale - BPRS-A, 5) State-Trait Anxiety Inventory - STAI, 6) Identification of the use of tobacco (issues selected from the Special Smoking Research "PETab", standardized protocol of the World Health Organization), 7) Fagerström Test for Nicotine Dependence - FTDN^11^, 8) Ladder Scale^12^ and 9) Modified Reasons for Smoking Scale - ERPFM^13^. 

The app was developed in partnership with the research group of the Health Intelligence Laboratory, consisting of professors and students of the Interunit Biomedical Informatics, of the School of Medicine of Ribeirão Preto (FMRP-USP) and the School of Philosophy, Sciences and Language of Ribeirão Preto (FFCLRP-USP). 

Between November and December 2013, planning meetings happened between the nurse researchers and the computer scientists. In this step, we defined the characteristics of the app and the resources that would be inserted, considering three criteria: 1) data security, 2) benefits for the patients (increased motivation and attention during the interviews) and 3) ease of use and optimization of the time of the researchers in the application of questionnaires. 

We decided to develop an app for mobile devices, named *TabacoQuest*, using the Android operating system as it is a platform distributed widely and freely by Google, in addition to the vast experience of the computer scientists in developing apps with this system.

Considering the extent and complexity of the questionnaires, we defined that the answers would be marked on the mobile device by the interviewer; however, the participants would follow their filling. So that participants could accompany the reading of the questions and their answers, we chose a device with a larger screen, with good resolution, containing high quality characteristics and long-lasting battery. We used the *Samsung Galaxy Note(r)* tablet, with 10.1 inch screen, Quad Core 1.4 Ghz, Android 4.0 operating system, 7000 mAh battery and 16 GB internal memory.

In the planning step, we defined that *TabacoQuest* would have three integrated functions: 1) data collection, 2) local storage in the native database of the device 3) data export for visualization and analysis (automatic generation of database). To this end, we predicted the need for a computer and a USB cable so that data could be transferred (exported) to the computer and viewed in an Excel spreadsheet. For the creation of the database and the Excel spreadsheet, the computer scientists followed a dictionary of variables developed under the guidance of a statistician. 

In January 2014, the first version of *TabacoQuest* was presented with the functions of data collection and database generation integrated. In order to test the accuracy of the answers marked in the tablet and the answers transferred to the database, twenty fictitious participants were created for testing. 

**Figure 1:**
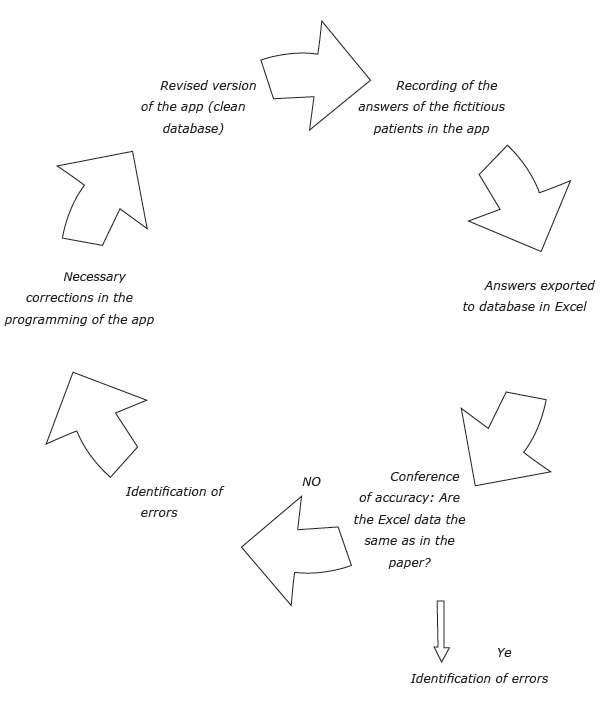
Testing process of TabacoQuest for data collection in research on mental health

Between January and February 2014, the answers of the fictitious participants, initially filled in printed questionnaires, were recorded several times in the app. Once transferred to the Excel spreadsheet, we analyzed if the answers of the database were the same as in the paper. For each error identified, the computer scientists carried out the necessary implementation corrections in the app and presented a new revised version. With the "clean" database, the answers of the twenty fictitious patients were recorded again, and then we verified the accuracy of the data. These procedures were repeated several times until all the bugs could be identified and corrected (Figure 1).

After the correction of the programming errors, in March 2014, we discussed the best strategies to improve the design of the *TabacoQuest*, in order to make it more attractive and friendly. In April 2014, the final version of the application was tested with six pilot participants, two from the mental health clinic, two from the psychiatric hospital and two from the basic health unit. The app was released for data collection after we and the computer scientists assessed its safety.

## Results

The results are presented in three topics: A) presentation of the app , B) main resources, purposes and advantages and C) limitations of the app.

### Presentation of the app

**Figure 2:**
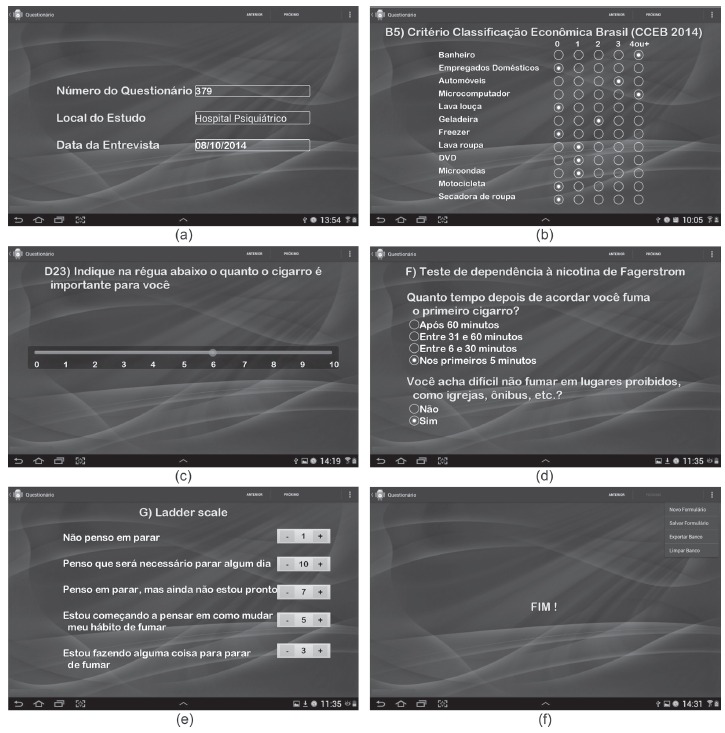
Screenshots of TabacoQuest: (a) - Automatic record of the number of the questionnaire and the date of the interview; (b) - Brazilian Economic Criteria; (c), (d) and (e) - Interaction: record of the answers using the touch screen; (f) - Indication of the end of the interview

In order to make the app attractive to the research participants, different interface options were contemplated. We eliminated the possibility of background with light color because of higher battery consumption. We opted for the interface that best highlighted the text (dark blue background), in addition to being attractive (Figure 2). We used *Arial Rounded MT Bold*, in yellow for the questions and words requiring emphasis and in white with shadow effect for the answers. In Figure 2 we show some *TabacoQuest* screenshots.

The initial screen of the app has functionality that automatically records the number of the questionnaire and the date of the interview (Figure 2a). On this screen, the nurse/researcher must enter the location of the study and select the "next" button to be directed to the following screen. 

We conceived and built a user-friendly interface, without excess of visual information or need for several touches in a single screen (Figure 2). The answers were all filled by the same nurse; however, in order to promote interaction with the interviewees, in some moments, they would be encouraged, with the supervision of the interviewer, to fill their own answer by touching the screen of the tablet (Figure 2c and 2e). The graphics were attractive and enabled interaction, thus providing better attention and motivation to the participants.

On the last screen of the app, a message appears indicating that the interview is done (Figure 2f). The nurse selects in the menu the option "save form" so that the answers can be stored in the native database of the tablet. To start a new interview, "new form" needs to be selected. To display the data, we need to select the option "export database" and all data stored on the tablet will be exported to an Excel spreadsheet. The connection is performed via an USB cable to the computer and then we can recover the file in Excel format, containing the data.

### Main resources, purposes and advantages

**Figure 3:**
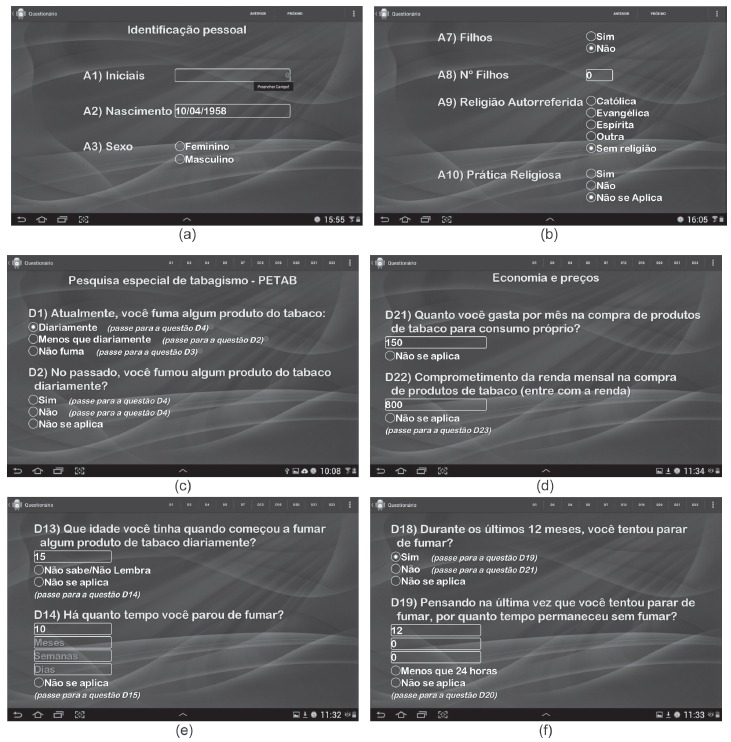
Screenshots with examples inserted in *TabacoQuest*: (a) and (c) - screenshots for validation of fields; (b) - branching logic: automatic response of question A8; (d) - calculator resource: the relationship between D21 and D22 is placed directly in the database; (e) and (f) - conversion of units

In Figure 3, we show some *TabacoQuest* screenshots that exemplify the resources in it. In order to avoid blank answers, the *field validation* feature has been inserted (Figure 3a and 3c). The app does not allow the interview to go on if any variable is not answered, thus ensuring the consistency and integrity of the data that will be stored. A red alert is displayed, indicating the need to select an alternative.

The questions are presented in the app according to the *branching logic*. In this way, the questions that are not applied to a particular participant are filled automatically or, in some cases, omitted. Figure 3b illustrates that, when the participant notes that he or she has no children, the next question (number of children) is registered automatically as zero. The same occurs with the variable "self-reported religion". If the participant has pointed out no religion, the variable "religious practice" is automatically registered as "not applicable".

Initially, we planned to apply the *branching logic* to all questionnaires. By examining the criteria for inclusion of resources, we admitted that although the *branching logic* could benefit participants (less tiring interviews) and optimize the time of the researcher, data security could not be guaranteed if this resource was applied in PETab questionnaires. 

The PETab is a complex questionnaire which presents conditions for all the answers (for example, if the answer is "daily" for question D1, we skip to question D4; if the answer was "less than daily" for question D1, we move on to question D2; if the answer is "never smoked" for question D1, we skip to question D3). We considered that it would not be safe the automatic guidance of the questions, which would then be conducted manually by the nurse/researcher. In order to guide the respondent through the interviews, statements have been inserted in front of each PETab answer, as well as shortcuts at the top of the app screen (Figure 3c). With this, if the nurse marks "daily" to question D1, there is a statement that the next question that must be answered is D4. The nurse/researcher clicks the shortcut "D4" at the top and is directed to the screen related to this question. In this case, the unanswered questions (D2 and D3) are automatically registered as "not applicable" in the database. 

In Figure 3d, we show the calculator resource. For example, in question D22, it is important to know how much the person spends on buying tobacco. Based on the value of the monthly income, answered in question D22, and the amount that is spent per month with the purchase of tobacco (inserted in question D21), the application automatically makes the calculation of the spending, registering it in the database. 

In figures 3e and 3f, we show examples of converting units of measurement. The answers to questions D14 and D19 must be recorded in the database as "days". However, the nurse/researcher has the option of inserting the answer as the participant gives them into the app. If the answer is in months, the number of months is inserted in the text box with the guidance "months". In the database, the answer will appear as "days".

Purposes and advantages of the resources inserted in *TabacoQuest* in order to facilitate and optimize the time of the interviews, increase the validity of responses and the interest of respondents:

- calculator - converts units of measurement. If the variable "how long have you stopped smoking" is recorded in the database in years, months or weeks, the calculator does the automatic conversion into days. In addition, it adds the total score obtained in the scales. It avoid miscalculations, increasing the accuracy of answers;
- timer - times the duration of the interview;
- offline operation - prevents interruption of interviews if the Internet signal stops working. It prevents loss of participants;
- user-friendly interface with interactive graphics - increases the interest and attention, providing a different interaction with the respondents. It makes the interview less tiring;
- branching logic - shows only the questions that apply to the respondent, in accordance with the previous answers. If it is recorded that the person is not a smoker, the variables specific to smokers are hidden. The questions that would be marked as "not applicable" are not asked, making the interview faster and less tiring. It avoids biases, increasing the accuracy of the answers;
- field validation - does not allow the continuation of the interview if a question is not answered, eliminating the possibility of blank responses. It does not allow the selection of more than one answer for questions with single answer, which would void the question. It ensures the consistency and integrity of the data stored;
- automatic tabulation - transfers the answers automatically to the Excel spreadsheet. It avoids typing errors and saves time for the researcher.

During the testing step, differences were identified in some calculations performed by the researchers in the printed forms of the fictitious patients and the answers recorded in the database. We noted that the calculation errors were committed by the researchers, and the app had registered the correct number, thus showing the accuracy of the answers and the potential to prevent human errors.

In addition to the benefits already mentioned, we also saw little difference in the cost that the researchers would have with the printing of the questionnaires and the cost of the tablet. 

### Limitations of the app

The main limitation of the app refers to its one-way operation. When initiated a new interview, the previous answers can no longer be retrieved (displayed) on the screen of the app. If there is the need to change any information, after the interview is saved, the researcher must perform it manually in the Excel spreadsheet. When an interview is interrupted, the researcher does not have the option to resume it another time, if a new form is started. When the interview is interrupted for a moment, if the researcher keeps the form open, it can be resumed; however, there is no option to pause the timer. In these cases, it is necessary to record, aside, the interruption time and then adjust the total time in the Excel database. 

## Discussion

When we thought about the possibility of a computerized data collection for research on smoking, we performed a search on the tools available. Although several options have been found, most required Internet connection at the time of the interview, were paid or did not have the resources necessary to address the complexity of some questionnaires used in the research. Therefore, we decided to develop an app.

For its development, we considered three criteria: 1) data security, 2) benefits for the participants (increased motivation and attention) and 3) ease of use and optimization of the time of the researchers in the application of questionnaires. The three issues could be met thanks to the partnership between health and science professionals, which allowed nurses to collaborate with their experiences of field work, prioritizing what could favor psychiatric patients and optimize the time of the research, and computer scientists with the knowledge on programming, ensuring the inclusion of safe resources that would not compromise the quality of the data.

It should be noted that, although the questionnaires are filled by the interviewer, we opted for a mobile device with a 10 inch screen to enhance interaction with research participants. In addition, when the individual showed an interest, he or she was encouraged to fill some of their answers on the screen of the device, with the supervision of the interviewer. 

Throughout the data collection with *TabacoQuest*, some patients approached the interviewer, driven by interest in knowing what she was doing in the service with the tablet and the contents of the app. Several persons offered themselves and even insisted on participating in the research. With this, we believe that the use of a mobile device on data collection can make persons more accessible and motivated to participate in the research, enabling the first contact with the researchers^4^.

Studies carried out in the United States (n=49), India (n=95) and Fiji (n=120) show that participants, including older ones, prefer to answer computerized questionnaires than answer printed questionnaires. For them, paper research requires more time, besides not being dynamic^3^ ^,^ ^8^ ^,^ ^14^.

A Swedish study has shown one of the main concerns regarding the use of mobile devices in the health field: the loss of the professional-patient interaction^6^. Similar to what occurred with the use of *TabacoQuest*, by reporting their experience with the use of Palm OS, for computerized data collection, a researcher said that the use of the mobile device favored the interaction with the patient and allowed greater eye contact than interviews conducted in paper^4^. The favoring of the visual contact was also reported in a Chinese study^15^.

Besides facilitating the visual contact between interviewer/interviewee and increasing the interest of persons in participating in the research, the computerized data collection has other advantages, depending on the resources that are included in the app: 1) data are validated at the time of the interview (which eliminates the possibility of blank answers or wrong selection of more than one answer), 2) it is not necessary to manually tabulate the data, thus decreasing potential human errors, 3) long questionnaires become less tiring (only the questions that apply to each participant are presented) and 4) data analysis can be started immediately after completing the number of participants, as it is not necessary to tabulate them^3^ ^,^ ^8^.

A study in Fiji, Oceania, has compared interviews recorded on paper and in a PDA (Personal Digital Assistant) device. Six persons were trained to interview 120 persons. Each person was interviewed twice, randomly, one with paper and one with the PDA. To assess the quality of the data, it was found that 20.8% of the printed questionnaires contained some type of error, while no computerized questionnaire presented errors^14^. In a systematic review of the scientific literature about the comparison between data collection on paper and on mobile devices, greater accuracy has been identified for the data collected on mobile devices in relation to paper^16^.

Given the current trend of using mobile devices for data collection of scientific research, it is important that nurses have computer knowledge, so that they can participate actively in the planning and preparation of technological tools to conduct their research, as well as in care management and assistance. In Brazil, the introductory computer education is provided for in the National Curriculum Guidelines of the Undergraduate Nursing Course^17^.

Our article brings an important contribution to nurses and other researchers by presenting every step of the planning of the app, the testing of its accuracy and the final version of *TabacoQuest*, with justification of the choice of each resource present in the app. This article can guide researchers from different areas of research who aim to develop apps for the first time, in addition to bringing a reflection on the importance of this digital resource in research studies with psychiatric patients.

Although the limitations of *TabacoQuest* have not harmed the research, we hope to develop alternatives to fix them. Future studies can compare the application of questionnaires in paper and using the tablet, in order to assess the acceptance of the app by the participants.

## Conclusions

The *TabacoQuest* was successfully developed, having shown, as its main potential, the stimulus for the involvement of persons with mental disorders and the general population in the research (it aroused curiosity in patients who were under care in the periods of data collection), the conduct of less tiring interviews (the user-friendly interface of the app favored greater interaction with the respondent and his or her participation in some answers), a bond between interviewer/interviewee favoring the trust and honesty of the answers (favoring greater visual contact) and the prevention of human errors with increased consistency and integrity of the data recorded (data validation at the time of the interview, automatic tabulation). 

The partnership between nurses and computer scientists in the development of *TabacoQuest* was essential to achieve the balance between app security, guarantee of benefits for the participants and optimization of the time of researchers. The success of this app as a research tool may encourage nurses to use this and other computational resources.
